# Systemic Administration of the Phytochemical, Myricetin, Attenuates the Excitability of Rat Nociceptive Secondary Trigeminal Neurons

**DOI:** 10.3390/molecules30051019

**Published:** 2025-02-23

**Authors:** Sana Yamaguchi, Risako Chida, Syogo Utugi, Yukito Sashide, Mamoru Takeda

**Affiliations:** Laboratory of Food and Physiological Sciences, Department of Life and Food Sciences, School of Life and Environmental Sciences, Azabu University, 1-17-71, Fuchinobe, Chuo-ku, Sagamihara 252-5201, Kanagawa, Japan; f22027@azabu-u.ac.jp (S.Y.); f22001@azabu-u.ac.jp (R.C.); f22015@azabu-u.ac.jp (S.U.); me2401@azabu-u.ac.jp (Y.S.)

**Keywords:** nociception, myricetin, trigeminal spinal nucleus caudalis, single-unit recording, CaV channel, Kv channel, pain management

## Abstract

While the modulation of the excitatory and inhibitory neuronal transmission by the phytochemical flavonoid, myricetin (MYR), has been noted in the nervous system, the way in which MYR affects the excitability of nociceptive sensory neurons in vivo remains to be established. This study aimed to explore whether administering MYR intravenously, in acute doses, to rats, diminishes the excitability of SpVc wide-dynamic range (WDR) spinal trigeminal nucleus caudalis (SpVc) neurons in response to nociceptive and non-nociceptive mechanical stimulation in vivo. Recordings of extracellular single units were obtained from SpVc neurons when orofacial mechanical stimulation was applied to anesthetized rats. The average firing rate of SpVc WDR neurons, to both non-noxious and noxious mechanical stimuli, was significantly and dose-dependently inhibited by MYR (1–5 mM, intravenously), and the maximum reversible inhibition of the discharge frequency, for both non-noxious and noxious mechanical stimuli, occurred within 5–10 min. The suppressive effects of MYR continued for about 20 min. These findings indicate that an acute, intravenous administration of MYR reduces the SpVc nociceptive transmission, likely through the inhibition of the CaV channels and by activating the Kv channels. Therefore, MYR might be utilized as a treatment for trigeminal nociceptive pain, without causing side effects.

## 1. Introduction

Signals from the orofacial area, conveyed by Aδ-fibers and C-fibers, pass through the trigeminal ganglion (TG) neurons to second-order neurons in the spinal trigeminal nucleus (SpV) [1]. The SpV caudalis (SpVc) acts as a significant relay center for nociceptive signal transmission [1,2]. There are two identified kinds of SpVc nociceptive neurons: the nociceptive-specific (NS) and the wide-dynamic range (WDR) neurons. Only the noxious stimuli affecting the receptive fields trigger responses in NS neurons, which might then transmit location-related details to superior centers [1,2]. The deep layer (IV-V) of WDR neurons receives input from both trigeminal primary nociceptive (Aδ-/C-) and non-nociceptive (Aβ-) afferents, that respond to both noxious and non-noxious stimulation [1,2]. When applied to the most sensitive region of the receptive field, graded nociceptive inputs lead to an increase in the firing frequency that corresponds to stimulus intensity, suggesting that WDR neurons encode stimulus intensity [1,2]. Since the previous studies have indicated that WDR neurons within the SpVc region are significant in hyperalgesia/allodynia and/or referred pain, these neurons frequently receive combined inputs from dental pulp, jaw, and masseter muscles, with notable changes in excitability after tissue damage [1,3,4].

Pain management often involves complementary alternative medicine (CAM) therapies, including acupuncture and herbal medicines, particularly when traditional Western medicine proves ineffective or raises concerns about side effects [5,6,7,8]. Earlier research has indicated that different dietary components can influence protective biological systems, including those related to cardiovascular and neural health, as well as anticancer systems [9,10]. Myricetin (MYR), a polyphenol member of the flavonoid group, is present in fruits and vegetables, and is recognized for its numerous beneficial biological effects, such as antioxidant, anti-inflammatory, and anticancer effects [11,12,13]. In vitro studies have demonstrated that MYR can inhibit the secretion of glutamate, an excitatory transmitter, in a concentration-dependent manner, through the suppression of voltage-gated calcium (CaV) channels in the nerve terminals of the cerebral cortex neurons [14]. In addition, MYR acts on the hypothalamic paraventricular neurons in vitro by promoting voltage-gated K (Kv) channels, thereby lowering the discharge frequency [15]. We have recently reported that the injection of one of the most common flavonoids, quercetin, into the peripheral receptive field, suppresses the excitability of nociceptive primary sensory neurons in the TG, possibly via the inhibition of voltage-gated Na (Nav) channels and opening Kv channels [16]. MYR also enhances the efficiency of the inhibitory synaptic transmitter, γ-aminobutyric acid (GABA), in the in vitro preparation of neurons of the central nervous system, by mechanisms occurring in both the presynaptic and postsynaptic membranes [17]. It has also been shown that MYR can cross the blood–brain barrier [18]. Collectively, these results indicate that systemically delivered MYR can cross the blood–brain barrier and reach the central nervous system, thus possibly inhibiting both CaV channels and excitatory glutamate transmission in the SpVc, strengthening the presynaptic inhibitory mechanism. However, the immediate impact of MYR on the trigeminal neuronal responses to both noxious and non-noxious mechanical stimuli in vivo has yet to be determined. Thus, the objective of this study was to examine if the acute intravenous administration of MYR to rats could reduce the excitability of nociceptive SpVc WDR neurons, in response to mechanical stimulation.

The present study provides evidence that, in the absence of inflammatory or neuropathic pain, acute intravenous administration of MYR produces a short-term inhibition of trigeminal nociceptive sensory transmission, possibly by inhibiting CaV channels and activating Kv channels. Therefore, MYR might be utilized as a treatment for trigeminal nociceptive pain without causing side effects.

## 2. Results

### 2.1. General Characteristics of SpVc WDR Neurons That Supply Trigeminal Nerve Fibers to the Facial Skin

**Figure 1:**
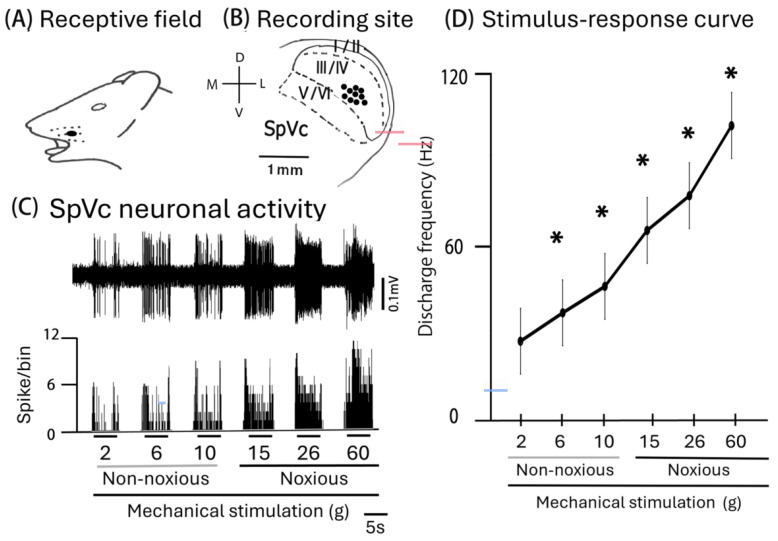
The general features of the spinal trigeminal nucleus caudalis (SpVc) wide-dynamic range (WDR) neuronal activity in response to mechanical stimulation. (**A**) Receptive field of the whisker pad in the facial skin (blackened area). (**B**) Localization of the layers I–IV in SpVc WDR neurons responding to both non-noxious and noxious mechanical stimuli on the facial skin (*n* = 10). (**C**) A standard illustration of the SpVc WDR neuronal responses triggered by non-noxious (2, 6, and 10 g) and noxious mechanical stimulation (15, 26, and 60 g) of the orofacial skin. Upper trace: SpVc WDR neuronal activity; lower trace: post-stimulus histogram. (**D**) SpVc WDR neuron stimulus-response characteristics (*n* = 10) * *p* < 0.05 for comparison of 2 g vs. 6 g, 10 g, 15 g, 26 g, and 60 g.

In this study, we recorded extracellular single-unit activity from 10 neurons in the SpVc, and the effect of the intravenously administered MYR was evaluated. A representative example of the receptive field of SpVc neurons, responding to both non-noxious and noxious mechanical stimulation in the whisker pad, is shown in Figure 1A, as described previously [19,20]. The main distribution of the recording sites was in the maxillary branches and layers III–IV in the SpVc (0.29 to 3.4 mm from the obex; Figure 1B). Examples of the SpVc WDR neuronal unit responses are depicted in Figure 1C. Graded mechanical stimulation was administered to the receptive field, leading to a proportional increase in the firing frequency of SpVc neurons. Two out of ten SpVc neurons showed spontaneous discharges. Figure 1D illustrates the correlation between mechanical stimulation intensity and average discharge frequency (stimulus-response curve) for WDR neurons. The average threshold for spike induction due to mechanical stimulation was 2.3 ± 1.7 g. All recorded neurons were classified as WDR neurons [20].

### 2.2. Influence of Intravenous MYR on the Excitability of SpVc WDR Neurons When Exposed to Non-Noxious and Noxious Stimuli

**Figure 2:**
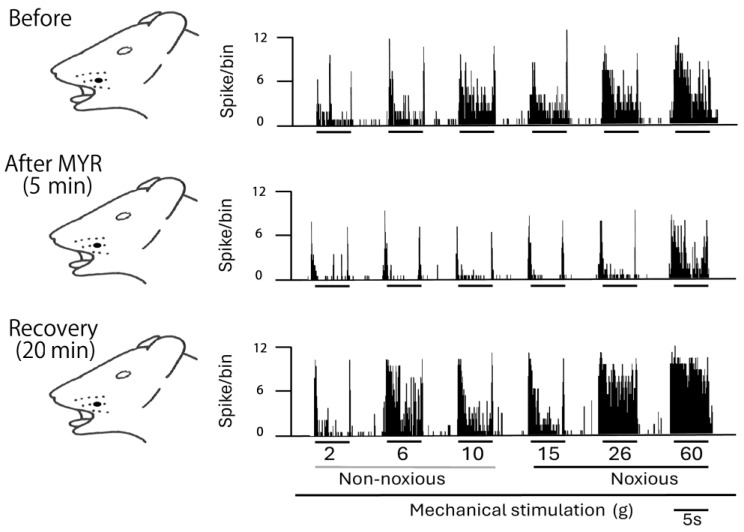
Influence of the intravenous 5 mM myricetin (MYR) on the neuronal activity of WDR neurons in the SpVc, evoked by non-noxious, noxious, and mechanical stimulation. Representative examples of the SpVc WDR neuronal activity, evoked by non-noxious (2, 6, and 10 g) and noxious (15, 26, and 60 g) mechanical stimulation: before, and 5 min and 20 min after the administration of 5 mM MYR. Blackened area indicates the location and size of the receptive field.

**Figure 3:**
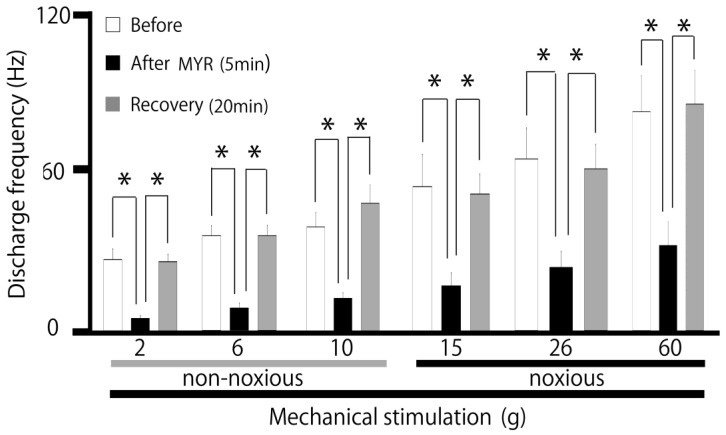
Time-dependent influence of intravenous 5 mM MYR on the average firing rate of the SpVc WDR neurons responding to non-noxious and noxious mechanical stimulation. * *p* < 0.05 before vs. 5 min after MYR; * *p* < 0.05, 5 min after MYR vs. recovery (20 min; *n* = 6).

**Figure 4:**
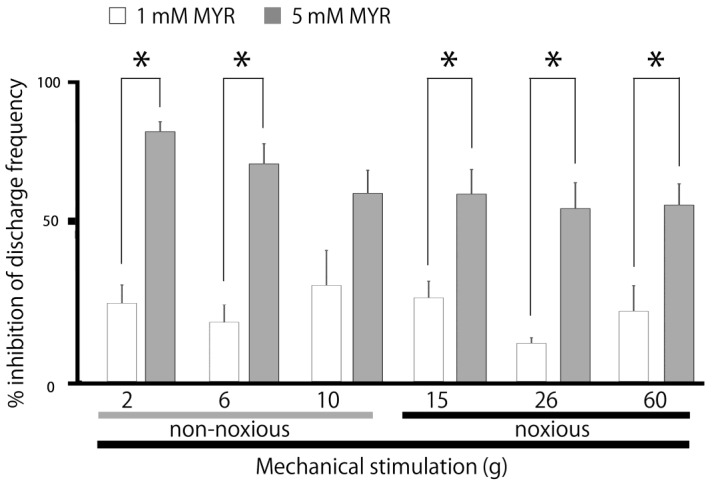
MYR induced a dose-dependent reduction in the average firing frequency of SpVc WDR neurons in response to both non-noxious and noxious mechanical stimuli. * *p* < 0.05, 1 mM MYR (*n* = 4) vs. 5 mM MYR (*n* = 6), i.v.

A typical example, shown in Figure 2, illustrates how the administration of intravenous MYR (5 mM) affects the excitability of SpVc WDR neurons in response to non-noxious stimuli. Five minutes after the intravenous injection of 5 mM MYR into the center of the receptive field non-noxious (2–10 g), mechanical stimulation-evoked SpVc WDR neuronal activity was inhibited, with activity returning to control levels within approximately 20 min. The size of the receptive field showed no notable differences before and after the MYR administration. No significant alterations in the mechanical threshold were noted following the MYR administration. The impact of MYR on the activity of SpVc WDR neurons triggered by non-noxious mechanical stimulation is summarized in Figure 3. After the injection of MYR, the mean firing rates of non-noxious mechanical stimulation-induced SpVc WDR neurons showed a significant reduction, compared to their rates prior to the injection (*p* < 0.05), and returned to control levels within 20 min (*p* < 0.05). No significant alterations in the spontaneous firing were detected following the intravenous injection of MYR. Figure 3 additionally illustrates typical instances of how an intravenous administration of 5 mM MYR affects the excitability of SpVc WDR neurons in reaction to noxious mechanical stimulation (15–60 g). The activity of SpVc WDR neurons induced by the noxious mechanical stimulation (15–60 g) was suppressed 5 min following MYR administration, but the neuronal activity returned to control levels within approximately 20 min (Figure 2). The average firing rates of SpVc WDR neurons, triggered by noxious mechanical stimulation, significantly declined following the MYR administration, as illustrated in Figure 3 (*p* < 0.05, *n* = 5). There were no significant changes in the size of the receptive field before and after the MYR administration. An intravenous injection of the vehicle (dimethyl sulfoxide) showed no significant influence on the spontaneous or mechanical stimulation-evoked activity of SpVc WDR neurons, regardless of whether the stimulation was noxious or non-noxious (*n* = 3), as described previously [19]. MYR (1–5 mM) showed a marked dose-dependent inhibition of SpVc WDR neuron firing in response to non-noxious mechanical stimulation (1 mM vs. 5 mM, *p* < 0.05; Figure 4). MYR (1–5 mM) also exhibited a significant dose-dependent suppression of noxious mechanical stimulation-evoked SpVc WDR neuron firing (1 mM vs. 5 mM, *p* < 0.05; Figure 4).

### 2.3. SpVc WDR Neuronal Activity in Response to Noxious vs. Non-Noxious Stimuli After MYR

**Figure 5:**
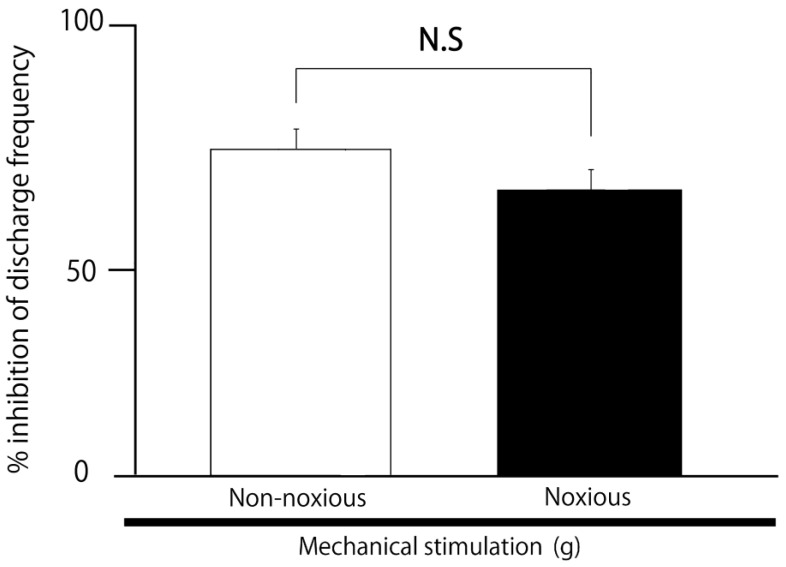
Comparison of the inhibitory effects of MYR on SpVc WDR neuronal discharge frequency under non-noxious and noxious stimulation. Not significant (N.S), non-noxious vs. noxious stimulation (*n* = 5).

We evaluated the comparative inhibitory effect of 5 mM intravenous MYR on reactions to non-noxious and noxious stimuli. The mean magnitude of the inhibition of discharge frequency caused by MYR was similar for both non-noxious and noxious stimuli (Figure 5).

## 3. Discussion

### 3.1. Acute Intravenous Administration of MYR Reduced the Excitability of SpVc WDR Neurons

The goal of this study was to examine if the acute intravenous administration of MYR to rats reduces the excitability of the SpVc WDR neurons in response to nociceptive and non-nociceptive mechanical stimulation in vivo. Here, we identified the following findings: *(i)* MYR (1–5 mM, i.v.) caused a dose-dependent reduction in the mean firing rate of SpVc WDR neurons, in response to both non-noxious and noxious mechanical stimuli; *(ii)* this inhibition of discharge returned to normal in approximately 20 min; and *(iii)* intravenous administration of a vehicle showed no notable impact on the spontaneous or evoked (non-noxious and noxious mechanical stimulation) responses of SpVc WDR neurons. A previous in vitro investigation found that 50 μM MYR significantly suppressed glutamate release from the preparations of rat cortical nerve terminals [14]. It is reasonable to conclude that following the intravenous delivery of 5 mM MYR, it would become diluted in the extracellular fluid, resulting in a particular concentration of approximately 0.05 mM (50 μM). We investigated the systemic administration of 5 mM MYR and its effects on SpVc WDR neuronal activity in this study and observed that this concentration had an impact on the nociceptive transmission of SpVc firing. Collectively, these results indicate that in vivo acute intravenous MYR administration inhibits trigeminal nociceptive transmission in the SpVc.

### 3.2. The Excitability of SpVc WDR Neurons Was Suppressed by MYR via Mechanisms at Both Peripheral and Central Levels

CaV channels open when the membrane depolarizes, permitting the entry of Ca^2+^ ions. This influx brings about two key consequences: *(i)* since Ca^2+^ ions possess two positive charges, the membrane undergoes additional depolarization, resulting in the activation of other voltage-gated ion channels (e.g., Na^+^ and K^+^ channels); and *(ii)* Ca^2+^ operates as a second messenger, facilitating the activation of multiple cellular functions, such as contraction, secretion, and gene transcription [21]. There are two types of voltage-gated Ca^2+^ channels: the low-voltage-activated (T-type) and high-voltage-activated (L, P/Q, N and R type) channels [22]. N-type and T-type CaV channels are involved in primary afferent signaling, particularly with high-voltage activated N-type channels being significantly concentrated at presynaptic terminals in laminae I and II of the dorsal horn [22,23,24]. Action potentials transmitted via dorsal root ganglion neurons, including C- and Aδ-afferents, initiate the opening of presynaptic N-type calcium channels, which in turn initiate the release of nociceptive transmitters, such as glutamate, substance P, and calcitonin-gene-related peptide, onto spinal interneurons and projection neurons [22]. A prior in vitro study indicated that MYR inhibited glutamate release from rat cortex nerve terminals in a dose-dependent manner by suppressing the presynaptic activity [14]. The same study also showed that N-, P-, and Q-type Ca^2+^ channel blockers eliminated the effect of MYR on the evoked glutamate release [14]. In this investigation, the average firing frequency of SpVc WDR neurons, in reaction to both non-noxious and noxious mechanical stimuli, was significantly and dose-dependently inhibited by MYR (1–5 mM, i.v.), and the maximum inhibition of the discharge frequency of both non-noxious and noxious mechanical stimuli was observed within 5–10 min. When considered together, it is likely that MYR delivered intravenously reduces the excitability of trigeminal nociceptive neurons via N-type Ca^2+^ channels, probably the presynaptic terminal of trigeminal ganglion (primary) neurons.

Kv channels contribute to numerous significant functions in the nervous system, including the regulation of the resting membrane potential, setting the action potential shape, neuronal repolarization, and neurotransmitter release via slow-inactivating sustained (K-current: I_K_) and fast-inactivating transient (A-current: I_A_) channels [25,26,27,28]. It has been previously observed that the reduction of I_A_ channel density, as opposed to I_K_ channel density, enhances the excitability of small-diameter neurons in rats in vivo [29]. Furthermore, the use of an A-type Kv channel blocker on TG neurons in vivo also boosted Aδ/C-TG neuronal activity, innervating the temporomandibular joint [30]. Consequently, an A-type Kv channel in Aδ/C-TG neurons innervating the temporomandibular joint is crucial for trigeminal inflammatory pain in temporomandibular joint disorders. Previous findings suggest that applying MYR lowers the discharge frequency by promoting Kv channels in vitro within hypothalamic paraventricular neurons [15]. We previously showed that the local application of chlorogenic acid, a polyphenol, reduced the discharge frequency of SpVc WDR neurons by opening Kv channels [19]. When Kv channels open, hyperpolarization of the resting membrane potentials is induced, thereby diminishing cell excitability, and several types of Kv channels have been proposed as targets for therapeutic approaches to pain management [29,30]. Overall, these results indicate that systemic MYR injection reduces the excitability of TG and SpVc neurons that respond to noxious mechanical stimulation, likely through the stimulation of Kv channels. Nonetheless, further in vitro studies are essential to assess this possibility.

**Figure 6:**
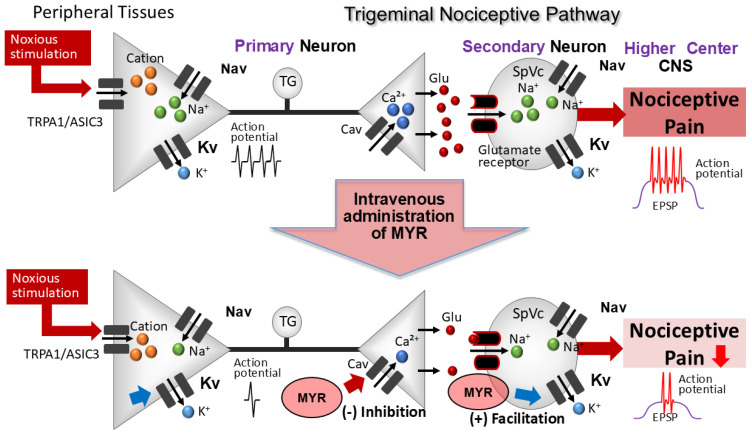
A potential mechanism for the inhibition of SpVc WDR neuronal discharge by MYR in response to nociceptive mechanical stimulation. When noxious mechanical stimulation is applied to the skin, mechanosensitive ion channels (transient receptor potential ankyrin 1 [TRPA1]/acid sensing ion channels 3 [ASIC3]) are activated, leading to the generation of a potential. This depolarization additionally activates voltage-gated sodium (Nav) and potassium (Kv) channels, leading to the generation of action potentials that are then conveyed through primary afferent fibers to the central terminal of nociceptive neurons in the SpVc. When the action potential arrives at the central end of the nerve terminal, voltage-gated calcium (CaV) channels in that area open, leading to a depolarization of the nerve terminal and permitting the entry of Ca^2+^ ions. When the intracellular concentration of Ca^2+^ rises, it prompts the discharge of excitatory neurotransmitters, such as glutamate (Glu), from the presynaptic neuron to the synaptic cleft. The elevation of intracellular Ca^2+^ levels stimulates the secretion of excitatory neurotransmitters, including Glu, from the presynaptic neuron, enabling cations to enter the cell by stimulating ionotropic secondary sensory neurons. When Glu receptors are stimulated, cations enter the cell, producing an excitatory postsynaptic potential (EPSP). Once this EPSP hits a defined membrane potential threshold, an action potential begins. The intravenous administration of MYR dampens the SpVc WDR neuronal excitability through the inhibition of CaV channels in the presynaptic terminal of the trigeminal ganglion (TG) neurons and post-synaptic glutamate receptors, leading to a decrease in the rate of action potential firing in SpVc WDR neurons that relay information to higher pain processing centers. CNS, central nervous system.

Zhang et al. [17] identified that MYR enhances GABA activity in neurons of the central nervous system in vitro, via mechanisms in both the presynaptic and postsynaptic membranes. In this study, we found that the MYR-induced maximal inhibition of the discharge frequency of SpVc WDR neurons, in response to both non-noxious and noxious mechanical stimuli, was reversible and occurred within approximately 20 min. Considering the short time course of the MYR effect on SpVc neuronal activity, it is reasonable to speculate that the inhibitory effect of nociceptive and non-nociceptive transmission evoked by acute MYR administration may not happen via a GABAergic mechanism. The fact that we found no significant change in the receptive field size before and after the MYR administration supports such a speculation, particularly in light of a previous study indicating that a local GABAergic mechanism in the SpVc modulates the mechanical receptive field properties [31]. Clearly, further studies are needed to identify the precise inhibitory mechanism at play, with nociceptive transmission in the presence of MYR. Finally, as summarized in Figure 6, we hypothesized the possible mechanism for the inhibition of SpVc WDR neuronal discharge by MYR in response to nociceptive mechanical stimulation.

### 3.3. The Significance of MYR’s Suppression of SpVc Neuron Excitability in Response to Nociceptive Stimulation

According to contemporary Western medicine, CAM is regarded as a medical system that has not undergone scientific evaluation or clinical use. Typically, CAM is well-known for its focus on herbal treatments and acupuncture. Recently, CAM has been regularly applied to patients with symptoms that Western medical approaches, including pharmacological treatments, fail to address, and it is expected to be useful in the treatment of chronic pain [5,6,7]. Prior investigations have found that various nutritional elements might affect mechanisms that offer biological protection, such as those in the cardiovascular, neural and anticancer systems [9,10].

Our previous research indicated that the phytochemical, resveratrol, may decrease the excitability of nociceptive neurons, under conditions of nociceptive and inflammatory pain in vivo, via voltage-gated ionic channels and transient receptor potential and ligand-gated channels [32]. For example, we have earlier indicated that a systemic delivery of resveratrol reduced inflammatory hyperalgesia, potentially through the cyclooxygenase-2 cascade [32]. Although our current study did not allow us to assess the effect of MYR on inflammatory pain, our findings indicate that the acute intravenous delivery of MYR reduces SpVc nociceptive transmission, possibly by inhibiting CaV channels and activating Kv channels, suggesting that MYR is a promising therapeutic option for alleviating trigeminal nociceptive pain, without causing adverse effects. MYR has been found to inhibit the production of cytokines, including prostaglandin E_2_ (PGE_2_), tumor necrosis factor-α, and interleukin-1β, which are generated in tissues during inflammation and amplify the inflammatory response [33], and also inhibits the production of the PGE_2_-producing enzyme, cyclooxygenase-2 [34,35]. PGE_2_ causes hyperalgesia by enhancing the sensitivity of mechanosensitive channels (transient receptor ankyrin 1/acid sensing ion channel) and voltage-dependent sodium and Kv channels through the activation of protein kinase A through prostanoid E-type receptors in pain-related terminals, while also lowering the activity of central glycine-sensitive inhibitory interneurons [32]. Recent studies have shown that MYR application mitigates neuropathic pain via the inhibition of CaV induced by p38 and protein kinase C [36]. In summary, these findings suggest that MYR lessens pathological pain, particularly inflammatory pain, by blocking the generation of inflammatory mediators and cytokines that are expressed in neurons. However, further investigation is essential to understand the mechanism that underlies MYR’s impact on trigeminal inflammatory pain.

## 4. Materials and Methods

Approval for the experiments described here was granted by the Animal Use and Care Committee at Azabu University (No. 200529-3) and adhered to the ethical guidelines set by the International Association for the Study of Pain [37]. Extensive actions were undertaken to decrease the use of animals and ease their suffering.

### 4.1. Extracellular Single-Unit Recording of WDR Neuronal Activity in the SpVc

Adult male Wistar rats (weighing 205–235 g) were kept under a fixed lighting schedule (lights on: 07:00–19:00). Temperature in the room was controlled, at 23 °C ± 1 °C. Food and water were available at all times. Recordings of electrophysiological activity were taken from 10 rats. Each rat was sedated with 3% isoflurane and maintained with additional doses of a mixture of anesthetics (0.3 mg/kg of medetomidine, 4.0 mg/kg of midazolam and 5.0 mg/kg of butorphanol) at 2–3 mg/kg/h, as required, through a cannula into the jugular vein. During the recording session, the absence of a reaction to paw pinching confirmed the level of anesthesia. A homeothermic blanket maintained the rectal temperature at 37.0 °C ± 0.5 °C (Temperature Controller, 40-90-8D; FHC, Aspen, Tokyo, Japan) during recording. Throughout the experiments, the edges of all wounds were consistently covered with a local anesthetic, 2% lidocaine (Xylocaine). The animals were subsequently situated in a stereotaxic device (SR-50; Narishige, Tokyo, Japan) and their neck muscles were divided along the animal’s midline. The atlanto-occipital ligament and the underlying dura mater were incised to access the medullary brain stem. A tungsten microelectrode (impedance 3–5 MΩ) was used to perform an extracellular recording of single-unit activity from the SpVc region in the ipsilateral medulla, and moved forward or backward in 10 μm increments using a micromanipulator (SM-11 and MO-10; Narishige), according to the stereotaxic coordinates of the rat brain atlas of Paxinos and Watson [20]. Neuronal signals were amplified (DAM80; World Precision Instruments, Sarasota, FL, USA), filtered (0.3–10 KHz), and observed using an oscilloscope (SS-7672; Iwatsu, Tokyo, Japan), and data were recorded for future analysis using PowerLab and Chart v.5 software (ADI Instruments, Oxford, UK) as described previously [19].

### 4.2. Experimental Protocols

The process for the analysis of extracellular single-unit SpVc WDR activity in response to whisker pad mechanical stimulation involved the following steps: To prevent sensitization of peripheral mechanoreceptors, the approximate receptor area of the receptive field in the left side of the whisker pad was swiftly identified using a paint brush as a search stimulus. Subsequently, the left side of the whisker pad was examined for individual units that reacted to a series of von Frey hairs (Semmes-Weinstein Monofilaments, North Coast Medical, Morgan Hill, CA, USA) with non-noxious (2, 6, and 10 g) and noxious (15, 26, and 60 g) mechanical stimulation for 5 sat intervals of 5 s [19]. The criteria for WDR neurons were previously identified as follows: graded mechanical stimulation, whether non-noxious or noxious, when applied to the receptive field, resulted in an increased firing frequency proportional to the stimulus strength. Following the identification of whisker pad-responsive nociceptive SpVc WDR neurons, we established the mechanical stimulation threshold and the size of the receptive field. The mapping of the neurons’ mechanical receptive fields was accomplished by applying von Frey hairs to the face and subsequently outlining them on a life-sized facial illustration [19]. The quantification of WDR neuronal discharges due to mechanical stimulation was achieved by removing the background activity from the evoked activity. Spontaneous discharge frequencies were determined over 2–5 min.

As previous research has shown that WDR neurons in the SpVc region play a crucial role in the mechanisms behind hyperalgesia and referred pain [1], our study focused on the influence of MYR on nociceptive SpVc WDR neuronal activity, omitting the examination of NS neurons. Post-stimulus histograms (bin = 100 ms) were generated in response to each stimulus [1]. The effects of intravenous administration of MYR (Sigma-Aldrich, Milano, Italy; 0.2 mL; 1 mM and 5 mM), through a Hamilton microsyringe, were evaluated 5, 10, 15, 20, 25, and 30 min after the administration, because the peak effect and recovery were thought to occur within this time-frame. MYR was dissolved in dimethyl sulfoxide to create a stock solution of 10 mM. The stock solution was stored at –20 °C until use. The stock solution was diluted to the desired concentrations using saline immediately before use. Mean spontaneous and mechanical stimulation-induced discharge rates, and the mechanical threshold before and after subcutaneous administration of MYR, were analyzed in the present study. The single-unit recording sites in the SpVc region were pinpointed using the micromanipulator, based on the distance from the obex, medial point, and depth from the surface of the medullary dorsal horn, with reference to the rat brain atlas, as described in our previous studies [19,20].

### 4.3. Data Analysis

Values are presented as the mean ± standard error. A one-way repeated-measures analysis of variance was used for statistical evaluation, with either Tukey–Kramer or Dunnett’s tests administered as post hoc analyses, and Student’s *t* tests for electrophysiological data. A two-sided *p*-value < 0.05 was considered to indicate a significant difference.

## 5. Conclusions

The current study presents the first evidence that, in the absence of inflammatory or neuropathic pain, rapid intravenous delivery of MYR leads to a short-term reduction in trigeminal sensory transmission, including nociception, potentially through the inhibition of CaV channels and the activation of Kv channels. Although further studies are needed to elucidate this mechanism, MYR might be viewed as a promising therapeutic candidate for alleviating trigeminal nociceptive pain without adverse effects.

## Data Availability

Data presented in this study are available on request from the corresponding author.

## References

[1] Iwata K., Takeda M., Oh S., Shinoda M., Farah C.S., Balasubramaniam R., McCullough M.J. (2017). Neurophysiology of Orofacial Pain. Contemporary Oral Medicine.

[2] Sessle B.J. (2005). Peripheral and central mechanisms of orofacial pain and their clinical correlates. Minerva Anestesiol..

[3] Shinoda M., Imamura Y., Hayashi Y., Noma N., Okada-Ogawa A., Hitomi S., Iwata K. (2021). Orofacial neuropathic pain-basic research and their clinical relevancies. Front. Mol. Neurosci..

[4] Sessle B.J. (2021). Chronic orofacial pain: Models, mechanisms, and Genetic and related environmental influences. Int. J. Mol. Sci..

[5] Rao J.K., Mihaliak K., Kroenke K., Bradley J., Tierney W.M., Weinberger M. (1999). Use of complementary therapies for arthritis among patients of rheumatologists. Ann. Int. Med..

[6] Konvicka J.J., Meyer T.A., McDavid A.J., Roberson C.R. (2008). Complementary/alternative medicine use among chronic pain clinic patients. J. Perianesthes. Nurs..

[7] Rosenberg E.I., Genao I., Chen I., Mechaber A.J., Wood J.A., Faselis C.J., Kurz J., Menon M., O’Rorke J., Panda M. (2008). Complementary and alternative medicine use by primary care patients with chronic pain. Pain Med..

[8] Bauer B., Tilburt C., Sood A., Li G.-X., Wang S.-H. (2016). Complementary and alternative medicine therapies for chronic pain. Chin. J. Integr. Med..

[9] Frémont L. (2000). Biological effects of resveratrol. Life Sci..

[10] Pervaiz S. (2003). Resveratrol: From grapevines to mammalian biology. FASEB J..

[11] Hakkinen S.H., Karenlampi S.O., Heinonen I.M., Mykkanen H.M., Torronen A.R. (1999). Content of the flavonols quercetin, myricetin, and kaempferol in 25 edible berries. J. Agric. Food Chem..

[12] Miean K.H., Mohamed S. (2001). Flavonoid (myricetin, quercetin, kaempferol, luteolin, and apigenin) content of edible tropical plants. J. Agric. Food Chem..

[13] Valdez L.B., Alvarez S., Zaobornyj T., Boveris A. (2004). Polyphenols and red wine as antioxidants against peroxynitrite and other oxidants. Biol. Res..

[14] Chang Y., Chang C.-Y., Wang S.-J., Huang S.-K. (2015). Myricetin inhibits the release of glutamate in rat cerebrocortical nerve terminals. J. Med. Food.

[15] Ma Z., Liu T. (2012). Myricetin facilitates potassium currents and inhibits neuronal activity of PVN neurons. Neurochem. Res..

[16] Toyota R., Itou H., Sashide Y., Takeda M. (2023). Suppression of the excitability of rat nociceptive primary sensory neurons following local administration of the phytochemical quercetin. J. Pain.

[17] Zhang X.H., Ma Z.G., Rowlands D.K., Gou Y.L., Fok K.L., Wong H.Y., Yu M.K., Tsang L.L., Mu L., Chen L. (2012). Flavonoid myricetin modulates GABAA receptor activity through activation of Ca2 + channels and CaMK-II pathway. Evid. Based Complement. Alternat. Med..

[18] Youdim K.A., Qaiser M.Z., Begley D.J., Rice-Evans C.A., Abbott N.J. (2004). Flavonoid permeability across an in situ model of the blood-brain barrier. Free Radic. Biol. Med..

[19] Kakita K., Tsubouchi H., Adachi M., Takehana S., Shimazu Y., Takeda M. (2018). Local subcutaneous injection of chlorogenic acid inhibits the nociceptive trigeminal spinal nucleus caudalis neurons in rats. Neurosci. Res..

[20] Paxinos G., Watson C. (1986). The Rat Brain in Stereotaxic Coordinates.

[21] Berridge M.J., Bootman M.D., Lipp P. (1998). Calcium- a life and death signal. Nature.

[22] Zamponi G.W., Lewis R.J., Todorovic S.M., Arneric S.P., Snutch T.P. (2009). Role of voltage-gated calcium channels in ascending pain pathways. Brain Res. Rev..

[23] Schaible H.G., Richter F. (2004). Pathophysiology of pain. Langenbecks Arch. Surg..

[24] Hildebrand M.E., Snutch T.P. (2006). Contributions of T-type Ca channels to the pathophysiology of pain signaling. Drug Discov. Today Dis. Mech..

[25] Ficke E., Heinemann U. (2001). Slow and fast transient potassium current in cultured rat hippocampus cells. J. Physiol..

[26] Hille B., Hille B. (2001). Potassium channels and chloride channels. Ion Channels of Excitable Membranes.

[27] Pearce R.J., Duchen M.R. (1994). Differential expression of membrane currents in dissociated mouse primary sensory neurons. Neuroscience.

[28] Lawson K. (2006). Potassium channels as targets for the management of pain. Cent. Nerv. Syst. Agents Med. Chem..

[29] Takeda M., Tanimoto T., Ikeda M., Nasu M., Kadoi J., Yoshida S., Matsumoto S. (2006). Enhanced excitability of rat trigeminal root ganglion neurons via decrease in A-type potassium currents following temporomandibular joint inflammation. Neuroscience.

[30] Hara N., Takeda M., Takahashi M., Matsumoto S. (2012). Iontophoretic application of an A-type potassium channel blocker to the trigeminal ganglion neurons enhances the excitability of Aδ- and C-neurons innervating the temporomandibular joint. Neurosci. Res..

[31] Takeda M., Tanimoto T., Matsumoto S. (2000). Change in mechanical receptive field properties induced by GABA_A_ receptor activation in the trigeminal spinal nucleus caudalis neurons in rats. Exp. Brain Res..

[32] Takeda M., Takehana S., Sekiguchi K., Kubota Y., Shimazu Y. (2016). Modulatory mechanism of nociceptive neuronal activity by dietary constituent resveratrol. Int. J. Mol. Sci..

[33] Jang J.-H., Lee S.-H., Jung K., Yoo H., Park G. (2020). Inhibitory effects of myricetin on lipopolysaccharide -induced neuroinflammation. Brain Sci..

[34] Rosas-Martinez M., Gutierrez-Vegas G. (2019). Myricetin inhibition of peptidoglycan-induced Cox-2 expression in H9c2 cardiomyocytes. Prev. Nutr. Food Sci..

[35] Gutierrez-Venegas G., Luna O.A., Arreguin-Cano J.A., Hernandez-Bermudez C. (2014). Myricetin blocks lipoteichoic acid-induced Cox-2 expression in human gingival fibroblasts. Cell Mol. Biol. Lett..

[36] Hagenacker T., Hileberand I., Wissmann A., Busselberg D., Schafers M. (2010). Anti-allodynic effect of the flavonoid myricetin in a rat model of neuropathic pain: Involvement of p38 and protein kinase C meditated Ca^2+^ channels. Eur. J. Pain.

[37] Zimmermann M. (1983). Ethical guidelines for investigations of experimental pain in conscious animals. Pain.

